# Optimising participation in a pulmonary rehabilitation programme for people living with chronic respiratory diseases in rural India: a feasibility study

**DOI:** 10.7189/jogh.15.04143

**Published:** 2025-05-09

**Authors:** Paul Jebaraj, Biswajit Paul, Rita Isaac, Shadrack Ravindra Reddy, Rakesh Kumar, Bochu Vikas, Deepa Das, John Norrie, David Weller, Hilary Pinnock, RESPIRE Collaboration

**Affiliations:** 1 Christian Medical College, Vellore, Rural Unit for Health and Social Affairs Department, Vellore, India; 2 Karkinos Healthcare Private limited, Ernakulam, India; 3 Bangalore Baptist Hospital, Bangalore, India; 4 Usher Institute, The University of Edinburgh, Edinburgh, UK

## Abstract

**Background:**

Pulmonary rehabilitation (PR) plays a vital role in managing chronic obstructive pulmonary disease and other chronic respiratory diseases (CRDs). However, implementation of this multidisciplinary approach in resource-poor settings may not be sufficient because of referrers’ uncertainty regarding the effectiveness of PR, inconvenient timing, travel issues, patients’ lack of motivation, and poor family support. The aim was to test the feasibility of a peer-led, professionally assisted community-based PR programme for CRD patients in a rural, low literacy setting.

**Methods:**

We conducted a single-centre, pre-post feasibility study. Participants with a confirmed diagnosis of CRD were recruited and treatment was optimised. After completing baseline assessments, the participants underwent eight weeks of PR training (16 sessions) in six groups at five local facilities led by peers selected by the participants and assisted by professionals. Exercise capacity was assessed with 6-Minute Walk Test. Other outcomes were: International Physical Activity Questionnaire; Hospital Anxiety and Depression Scale; London Chest Activity of Daily Living scale. Upper and lower limb strength were assessed using a handheld dynamometer and cycle ergometer, respectively. Descriptive analysis was performed, and pre-and post-outcomes were compared using parametric tests.

**Results:**

Thirty participants (20 chronic obstructive pulmonary disease and 10 asthma; 15 female; median age 57.5 years) completed baseline and endline assessments. Seventy percent completed at least 12/16 sessions. After eight weeks of training, the 6-Minute Walk Test had improved from 263.3 (standard deviation (SD) = 72.3) to 319.6 (SD = 84.7) metres (*P* < 0.001) with significant improvement in modified Medical Research Council (*P* = 0.022), London Chest Activity of Daily Living scale (*P* < 0.001) and dominant handgrip strength (*P* < 0.001) but no significant change in physical activity (*P* = 0.791).

**Conclusions:**

The community-based PR led by peer volunteers and supported by professionals proved to be feasible in our low-resource setting and was associated with improved exercise tolerance and other outcomes.

**Registration:**

The study was registered at the Clinical Trials Registry – India (CTRI/2020/09/027818).

Chronic respiratory diseases (CRDs) are a group of heterogeneous disorders that develop gradually, persist over time [1], and globally account for nearly a third of deaths [2]. In India, the most prevalent CRDs include chronic obstructive pulmonary disease (COPD), which has a prevalence of 8% (95% confidence interval (CI) = 6–9). This is followed by asthma, which exhibits prevalence between 3.3–8.7% (95% CI = 5.1–13.1) [1]. Chronic respiratory diseases associated with dyspnoea, fatigue, malnutrition, and diminished strength and endurance adversely affects a patient’s health-related quality-of-life [3,4]. Medications have a role in improving residual disability, functionality, and dependence [5] but do not restore normal functioning. The Global Initiative for Chronic Obstructive Lung Disease guidelines recommend pulmonary rehabilitation (PR), a supervised programme that combines exercise (both endurance and muscle-strengthening exercises) and education (encompassing causes and treatment of CRDs; dietary advice; awareness and self-management; psychological and social support) to reduce dyspnoea and fatigue, increase exercise tolerance and improve functionality, and improve health-related quality-of-life [6–9] for people with CRD. This tailored supervised programme involves a pulmonologist, physiotherapists, occupational therapists, psychologists and nutritionists. The benefit of PR has been well-established in both COPD and asthma patients [10–14], however, globally only 1–2% of CRD patients have access to PR services [15].

In India, access to PR services is a challenge for CRD patients as there are barriers at the level of health care systems, professionals, and patients [16]. It is important to identify optimal ways to enable CRD patients to benefit from PR services by overcoming those barriers in resource limited settings for better outcomes. Pulmonary rehabilitation can be effectively implemented in resource limited rehabilitation settings through nurse-led and student-led PR programmes [17,18]. In India, there is evidence that community volunteers or peers can deliver health care services in remote areas [19,20]. This paper presents the findings from a feasibility study implementing peripheral centre-based, peer-led, professional-assisted, comprehensive PR in a rural Indian setting and the associated impact on the well-being of patients.

## METHODS

### Study design

Aligned with the developmental phases of the Medical Research Council Framework for developing and evaluating complex interventions [21] we designed a prospective, quasi-experimental, single-centre, pre-post feasibility study.

### Study setting

The study was conducted from February–July 2021 in the K.V.Kuppam block, a rural development block (a sub-division of a district in India with more than 100 000 people) in the Vellore district, Tamil Nadu, South India. The majority of the population is involved in agriculture and 30% of them live below the poverty line. The literacy rate among men is 77%, and that among women is 64%. Two thirds of the population use biomass fuel for cooking. A secondary-level hospital, Rural Unit for Health and Social Affairs (RUHSA) Department, Christian Medical College, Vellore provides primary care services through a network of local volunteers (community health workers) along with medical staff in the study area. The local prevalence of asthma was 16.3%, that of COPD was 4.5%, and that of ‘other chronic respiratory disease’ was 3.0% according to a previous study [22]. Rural Unit for Health and Social Affairs (RUHSA) is a partner of the UK National Institute for Health and Care Research (NIHR)-funded Global Health Research Unit on Respiratory Health (RESPIRE) [23] offering the opportunity to develop and test a PR service.

### Study participants

The participants were recruited from the intervention arm of the ‘RESPIRE-PDT’ (The NIHR RESPIRE – Prevention, Detection and Treatment) randomised trial [23]. The RESPIRE-PDT study developed an innovative community-based programme to raise awareness of CRD, and tested an intervention that could be implemented in the local population to improve CRD outcomes, and understand adherence to CRD treatment in poor, rural populations in India. Using the RESPIRE respiratory questionnaire [22], door-to-door screening was performed by trained community health workers to identify participants (adults ≥18 years of age) with symptoms of CRD disease in four randomly selected clusters out of 18 in the study area. Screened positive participants were invited to the RUHSA hospital for baseline assessment and lung function tests. Those with confirmed diagnoses of CRD were provided information about the study, and those who provided consent were recruited into the study. All participants were provided with inhalers and general education on CRD and its management on the basis of health belief model. Four clusters were randomly allocated equally (2:2) to the intervention and control arms with 100 participants in each arm. The participants in the intervention arm received health education on the steps of inhaler usage and breathing exercises, and group and family education using short videos based on the Theory of Planned Behaviour [24] once in every three months by the trained community health worker. Chronic respiratory disease clinic was set at the base hospital and participants were invited to attend the clinic once in three months for one year. At the end of one year, endline assessment and spirometry were completed. Participants in the intervention arm were assessed for eligibility (**Table 1**) by the study physician and were referred to the study co-ordinator of the current study. The participants who were not adherent to treatment were again reassessed and were provided with inhalers (treatment optimised).

**Table 1:** Inclusion and exclusion criteria for participants and peer volunteers

Participant and peers	Criteria
Inclusion criteria for participants	mMRC dyspnoea grade of two or more for COPD
	Asthmatics with step two and above based on Global Initiative for Asthma guidelines
	PFT showing a FVC<65%, or FEV_1_<65%
	Having adequate cognitive ability
	Willing to participate in the study
Exclusion criteria for participants	Any diagnosis with angina pectoris, recent myocardial infarction, severe pulmonary hypertension, congestive heart failure, unstable diabetes, any psychiatric illness and severe exercise-induced hypoxemia
	Inability to do exercise due to orthopaedic or other reasons
	Not correctable with O_2_ supplementation
Inclusion criteria for peer volunteers	Age above 18 years
	Able to commit 6–8 hours in a week for eight weeks of PR training programme
	Willing to visit the participant’s home
	Have a good understanding on impact of CRD in the community
	Consented to take part in the study
Exclusion criteria for peer volunteers	Current smokers or addicted to alcohol
	Having any psychiatry illness, or any diagnosis with angina pectoris, recent myocardial infarction, severe pulmonary hypertension, congestive heart failure, unstable diabetes
	Inability to do exercise due to orthopaedic or other reasons.

COPD – Chronic Obstructive Pulmonary Disease, CRD – Chronic Respiratory Disease, FEV_1_ – forced expiratory volume in one second, FVC – forced vital capacity, mMRC - modified Medical Research Council, O_2_ – Oxygen, PFT – Pulmonary function test, PR – pulmonary rehabilitation

### PR assessments and data collection

Eligible participants were provided with the information sheet and an audio-recording of the information sheet was played before providing written informed consent. Following consent, the study physician took a detailed medical history, smoking history, treatment history, and socioeconomic assessment. Pulmonary function tests, haemoglobin levels, and body mass index were performed. Trained interviewers assessed physical activity level using the International Physical Activity Questionnaire, anxiety and depression using the Hospital Anxiety and Depression scale (HADS), and functional capacity using the London Chest Activity of Daily Living scale. Exercise capacity was assessed using the 6-Minute Walk Test (6-MWT) by a trained physiotherapist according to the American Thoracic Society (ATS) guidelines [25]. Upper and lower limb strength were assessed using the handheld dynamometer and cycle ergometer, respectively. The initial assessment took 2–3 days to complete and those who completed the assessment were recruited into the study.

### Selection and training of peer volunteers (PVs)

The recruited participants, community members, and leaders selected the peer volunteers (PVs) and peripheral centre for PR during the patient and public involvement meeting conducted at the base hospital. The selection criteria for PVs are provided in **Table 1**. The selected volunteers were trained by professionals (occupational therapist and a physiotherapist) through a two-day workshop. The workshop focused on the role of a peer supporter, training on the exercises that participants would be doing during the PR programme, how to assist participants in writing their exercise logbook, recording day-to-day activities performed in a diary, and ways to motivate participants to comply with the PR programme.

### Intervention: eight-week peripheral centre-based comprehensive PR programme

The participants attended a structured, eight weeks pulmonary rehabilitation training programme that was conducted in five peripheral centres in the community with six participants in each centre. The exercise training sessions (Table S1 in the **Online Supplementary Document**) were conducted for two hours a day, twice a week for eight weeks (16 sessions). The first four weeks of PR training were led by professionals and the remaining four weeks were led by PVs with assistance from professionals. This task-shifting was a strategy for sustainability. The PVs were supported to take responsibility for organising supervised exercise training sessions which they could maintain after a completed pulmonary rehabilitation programme [26].

All participants were provided with a pictorial booklet on recommended exercises and a 20-minute therapist-led exercise video clip was transferred to their or their family member’s smartphones to use during the unsupervised sessions. The participants recorded the exercises in their logbooks, and if illiterate, were assisted by the PVs. Similarly, the PVs asked about the number of unsupervised PR sessions per week, any barriers to exercising at home, and any other relevant issues which they recorded in the diary during their visit to the participant’s home. The exercise logbook and PV’s diary were reviewed by the research supervisors weekly to monitor progress with unsupervised exercise sessions. An average of 3–4 participants was supported by one PV.

The nutritionist educated the undernourished, obese, and anaemic participants and provided a diet plan on the basis of the 24-hour dietary recall assessment. Group sessions along with educational hand-out materials were conducted in the local language during the first week, and an education session was conducted during the fourth week of PR training at the centre. The psychologist provided individual, family, and group counselling to those who had a score of 8–10 on the HADS scale at the centre and those with a score of 10 and above were referred to a psychiatrist for diagnosis and treatment at the base hospital. Four individual counselling sessions were conducted in the first four weeks of PR training in the centre by the psychologist. The psychologist used a therapeutic storytelling technique based on narrative therapy [27], a respectful, non-blaming approach to help participants examine their life stories from a different perspective during the counselling sessions. Individual sessions focused on reinterpreting participant’s experiences and challenging negative beliefs. Other interventions included relaxation techniques, strengthening personal coping skills, making aware of their issues, taking ownership of their issues, motivation, and positive reinforcements. Group and family sessions were conducted in the 5th and 6th week to facilitate family and social support, improve decision-making skills, and raise awareness that they are not alone in facing mental health problems.

All centres followed strict guidelines as per the Indian Council for Medical Research (ICMR) COVID-19 prevention protocol [28], including the use of a surgical mask, frequent hand washing using sanitizers, and social distancing while exercising. No more than six participants were allowed at a time in a hall of 1000 square feet size. After completing eight weeks of training, within two weeks, all the participants completed the endline assessment at the base hospital.

### Process evaluation

A process evaluation was undertaken by an independent research team to explore the feasibility of PR implementation in a rural setting. Qualitative research methods such as observation and short structured interviews were used to assess participant’s perceptions and satisfaction with the PR programme, and the involvement of PVs and professionals. The evaluation was performed at the 2nd, 6th and 8th week of the PR programme.

### Statistical analyses

Descriptive statistics are reported as the means (standard deviations) and medians or frequencies for demographics, clinical characteristics, and outcomes. Paired *t* test for continuous variables and χ^2^ test for categorical variables were performed to compare pre-post-PR outcomes. The level of significance was set at a two-sided *P* < 0.05 for all comparisons. Data was analysed using IBM SPSS Statistics for Windows, Version 21.0 (IBM Corp., Armonk, NY, USA).

## RESULTS

**Figure 1:**
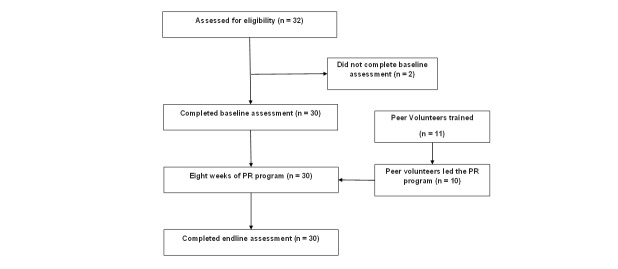
Study flow diagram as per CONSORT (Consolidated Standards of Reporting Trials) guidelines. PR – pulmonary rehabilitation.

Out of 100 participants, 46 were eligible, and 32 consented to participate in the study. Finally, 30 participants completed the baseline assessment (**Figure 1**) and were recruited to participate in the study (**Table 2**). Eleven PVs consented to support the participants.

**Table 2:** Sociodemographic and clinical characteristics of the participants at baseline*

Sociodemographic and clinical characteristics	Total (n = 30)
Age in years, MD (IQR)	57.5 (47.5–70.25)
Female	15 (50)
BMI	
*Severe under nutrition (<16)*	1 (3.3)
*Mild to moderate under nutrition (16–18.49)*	2 (6.7)
*Normal (18.5–24.9)*	12 (40)
*Over weight (25–29.9)*	11 (36.7)
*Obesity (>30)*	4 (13.3)
Anaemia	
*Male (<13 g/dL)*	4 (26.7)
*Female (<12 g/dL)*	6 (40)
Smoking status	
*Never*	26 (86.6)
*Former smoker*	2 (6.7)
*Current smoker*	2 (6.7)
Living Status	
*Alone*	3 (10)
*With partner and children*	27 (90)
Diagnosis	
*Bronchial Asthma*	10 (33.3)
*COPD*	20 (66.7)
COPD disease grade based on GOLD	
*mMRC grade 2*	8 (30)
*mMRC grade 3*	10 (50)
*mMRC grade 4*	2 (10)
Asthma disease severity	
*GINA Step 2 treatment*	4 (40)
*GINA Step 3 treatment*	3 (30)
*GINA Step 4 or 5 treatment*	3 (30)
Pulmonary Function (post bronchodilator)	
*FEV_1_ (L), x̄ (SD)*	1.28 (0.46)
*FEV_1%_ pred, x̄ (SD)*	64.03 (27.27)
*FVC (L), x̄ (SD)*	2.17 (0.53)
*FVC % pred, x̄ (SD)*	87.37 (21.89)
*FEV_1_/FVC (ratio), x̄ (SD)*	0.60 (0.16)
*FEV_1_/FVC% pred, x̄ (SD)*	73.63 (18.65)
Depression (HADS screening score)	
*Borderline depression (8–10)*	8 (26.7)
*Depression (11–21)*	6 (20)
Anxiety (HADS screening score)	
*Borderline anxiety (8–10)*	9 (30)
*Anxiety (11–21)*	6 (20)

BMI – body mass index, COPD – chronic obstructive pulmonary disease, FEV_1_ – forced expiratory volume in one second, FEV_1_% pred – forced expiratory volume in one second/estimated value of FEV_1_, FVC – forced vital capacity, FVC% pred – predicted forced vital capacity, GINA – Global Initiative for Asthma, GOLD – Global initiative for chronic obstructive pulmonary disease, HADS – Hospital Anxiety and Depression Scale, MD (IQR) – Median (interquartile range), mMRC – modified medical research council dyspnoea scale, SD – standard deviation, x̄ – mean, L – litres

*Data presented as n (%) unless otherwise specified.

Seventy percent of the participants completed eight weeks of the PR programme (12 out of 16 sessions). After eight weeks of training, there was a significant improvement in the 6-MWT in meters (*P* = 0.001), the dyspnoea level (*P* = 0.022), the exercise endurance time with a constant-load of 80% of maximum work rate, Wmax (*P* ≤ 0.001), the handgrip strength of the dominant hand (*P* ≤ 0.001), the % total score of London Chest Activity of Daily Living (*P* ≤ 0.001), the Body mass index, Obstruction (airflow), Dyspnoea, and Exercise capacity for COPD Survival (*P* ≤ 0.001) and the HADS score for anxiety and depression (*P* ≤ 0.001). No significant differences were observed in the physical activity (*P* = 0.791) and the following pulmonary function measures: forced expiratory volume in one second (FEV_1_) litres (L) (*P* = 0.385), % of predicted FEV_1_ (*P* = 0.977), forced vital capacity (FVC) L (*P* = 0.078), % of predicted FVC (*P* = 0.166) and % of predicted FEV_1_/FVC (*P* = 0.551) at the end of eight weeks of PR (**Table 3**).

**Table 3:** Change in outcomes after eight weeks of pulmonary rehabilitation

Outcome variables	N	Baseline x̄ (SD)	Endline x̄ (SD)	*P-*value
6-MWT (distance in metres)*	30	263.3 (72.3)	319.6 (84.7)	<0.001
Modified mBorg Dyspnea Scale (0–10)*	30	3.7 (1.8)	2.8 (1.6)	0.022
CET (Work Rate mean in watts)*	30	72.1 (28.7)	73.6 (33.3)	0.766
Endurance cycling (time in seconds)*	30	104.1 (58.5)	168.7 (74.4)	<0.001
Hand grip strength of dominant hand (kilogrammes), x̄ (SD)*	30	15.2 (4.7)	20.3 (5.7)	<0.001
IPAQ (MET minutes per week)*	30	4585.5 (4300.4)	3148.1 (3319.6)	0.43
LCADL total (0–75 points, the highest score indicating the greatest incapacity to perform ADLs)*	30	26.9 (12.3)	22.10 (11.3)	0.092
LCADL total %*	30	39.5 (14.3)	31.2 (14.1)	<0.001
BODE index for COPD survival*	20	4.9 (2.4)	2.3 (2.2)	<0.001
HADS depression*	30	7.1(4.6)	2.1(2.8)	<0.001
HADS anxiety*	30	7.5(4.9)	3.4(3)	<0.001
HADS total score*	30	14.7(9.2)	5.4(5.6)	<0.001
Physical activity*	30			0.791
*High activity (>1500 MET-min/week)*		19 (63.3)	18 (60)	
*Low & moderate activity (≤1500 MET-min/week)*		11 (36.7)	12 (40)	
COPD disease grade based on GOLD	20			
*mMRC grade 1*		0 (0)	8 (40)	
*mMRC grade 2*		8 (40)	6 (30)	
*mMRC grade 3*		10 (50)	4 (20)	
*mMRC grade 4*		2 (10)	2 (10)	
Asthma disease severity	10			
*GINA Step 1 treatment*		0 (0)	1 (10)	
*GINA Step 2 treatment*		4 (40)	7 (70)	
*GINA Step 3 treatment*		3 (30)	2 (20)	
*GINA Step 4 or 5 treatment*		3 (30)	0 (0)	

6-MWT – 6-Minutes’ walk test, ADL – Activity of daily living, BODE – Body mass index, Obstruction (airflow), Dyspnoea, and Exercise capacity, CET – Cycle endurance test, COPD – chronic obstructive pulmonary disease, GINA – Global Initiative for Asthma, GOLD – Global initiative for chronic obstructive pulmonary disease, HADS – Hospital Anxiety and Depression Scale, LCADL total – London Chest Activity of Daily Living, LCADL total% – % of total London Chest Activity of Daily Living, MET – Metabolic Equivalent of Task, mMRC – modified Medical Research Council dyspnoea scale, N – Number of participants, IPAQ – International Physical Activity Questionnaires, SD – standard deviation, x̄ – mean

*Paired *t* test.

### Insights into the operational feasibility of PR

A total of 28 in-depth interviews with the participants, six semi structured interviews with PVs, and 10 peer-led, professional assisted training sessions were conducted by an independent research team. The median interview time was 60 minutes (range = 30–120); most interviews took place at the centre and few occurred at the participants’ homes. The subthemes identified were as follows below.

### PR experience and its impact

The participants reported that it was their first time exercising for two months and that it motivated them to continue in their homes. They also reported that the six peripheral centres chosen were at a walkable distance from home; the training environment was quiet and private; and open corridors, balconies, or a small open space adjacent to their house was used for home sessions. Most of the participants felt better after doing the exercises. They reported that breathing exercise eased their breathing problems and reduced wheezing. One of the participants stated:

*Regularly practicing the exercises has improved my quality of life as my breathing has improved.*

The participants also reported that their general health improved after exercising. They reported reduced back pain, felt stronger than before, and felt that they needed to improve their physical fitness:

*I feel strong after doing exercises. Need to improve my walking, so, it will not affect my livelihood.*

### Opinion on the role of peer volunteers in PR

The participants felt that the PVs were supportive, caring and encouraging. The PVs were largely active and involved in teaching exercises. They were sensitive to the participant’s health conditions and encouraged them to exercises at their own pace. Compared to men, women volunteers showed greater personal involvement and empathy for the patients.

With respect to the increase in PVs, the participants paid attention to the PVs instructions. However, negative remarks were expressed on home visits by the PV, such as the following:

*He (PV) coming to my home has led my neighbours talk negatively about me as a ‘diseased person,’ I was uncomfortable due to stigma and therefore requested him not to come home.*

### Difficulties in adhering to PR training

The participants who missed the PR sessions reported exacerbation as a major reason, followed by worsening of coexisting conditions and other priorities (attending a funeral or wedding). They also reported that they did not understand the exercises, especially ‘purse lib breathing,’ and that a few exercises were difficult to perform (sit-ups):

*Thighs and knees hurt after doing exercises. I had to give hot water fomentation and then felt better. Had to take leave from work twice a week but despite this it is worth it.*

However, most took this as a challenge and attended training sessions with determination to improve their health condition. Those who were not able to perform the exercise regularly at the centre were keen to return when they were able.

## DISCUSSION

Pulmonary rehabilitation is recommended as the standard of care for CRD patients despite pharmacological therapy [29,30]. The concept of PR was introduced in India in 1990 and it is known that PR reduces the overuse of health care resources [31] in CRD management. Despite studies showing good clinical outcomes of successfully implementing PR in resource-limited settings [32–35], potential barriers still exist [16]. Owing to the lack of trained personnel [36], clinicians are not motivated to include PR as part of CRD treatment in their crowded outpatient departments. The limited local availability of established pulmonary rehabilitation facilities is a systems barrier which discourages health care professionals and makes attending centre-based sessions impractical for patients [36]. Other recognised factors include patient preference for medications over non-pharmacological therapy, poor family and social support, unavailability of PR services close to their homes at a convenient time, restricted mobility, and poor knowledge of the benefits of PR [16,37].

This pre-post feasibility trial addressed these barriers by educating the community, family, and participants, and providing community support through trained PVs, peripheral PR centres for better access, and skilled therapists to monitor the training. The results were promising in that 70% of the participants completed eight weeks of training, similar to attending centre-based PR in an urban setting. The functional capacity of the CRD patients improved by 56 m in the 6-MWT at the end of eight weeks of training similar to other PR studies [38,39]. This significant improvement in the % total score of activity of daily living which is consistent with the finding of previous studies and has a better correlation with clinical outcomes [40] at the end of the eight-week PR programme. However, this study did not find an apparent improvement in lung function following rehabilitation, as reported in other studies [41].

The HADS baseline score (**Table 2**) indicated the presence of anxiety and depression symptoms among our study participants similar to the findings of other studies [42]. The minimal clinically important difference for HADS is approximately 1.5 for CRD patients [43], and the mean change in the participants after the 8-week programme exceeded this (**Table 3**). Compared with age-matched controls, CRD patients have reduced upper and lower muscle strength and endurance due to the deconditioning of skeletal muscles as [44]. We observed significant changes in upper limb strength and lower limb endurance (**Table 3**) measured with handheld dynamometer and cycle endurance test, respectively, but no significant change in physical activity. Changing the sedentary habits of a lifetime reinforced by the uncomfortable symptoms of breathlessness is challenging, but the presence of local PVs may support behaviour change over time. The endline assessment was conducted in the winter which may have been another factor. Feasibility studies are not powered to deliver statistically significant results which mean that true differences may have been missed.

Our study was designed to fit within the developmental phases of the Medical Research Council Framework for developing and evaluating complex interventions [21]. Our priority was to establish the feasibility of our novel approach to delivering pulmonary rehabilitation. The pre-post design enabled us to learn from the delivery of the intervention to more participants than if we had undertaken a pilot randomised controlled trial, but we recognise than a non-randomised design precludes any inference of effectiveness. Strength of our design is that the qualitative study was undertaken by an independent research team that was not part of the study which increases the internal validity of the study.

## CONCLUSIONS

The associations demonstrated in our study are encouraging, but further research is needed to establish the effectiveness. Informed by our feasibility work, the Pulmonary Rehabilitation trial will be commencing soon in four Centres in South/South East Asia [45]. In the meantime, community-based pulmonary rehabilitation led by community volunteers with the assistance of professionals is a feasible option for implementing local services for people with CRD in low-resource settings. The National Programme for Prevention and Control of Non-Communicable Diseases launched by the Government of India in 2010 recommends early detection, medical management, appropriate referrals, and a continuum of care for patients with COPD and asthma [46]. Our findings support the inclusion in the National Programme for Prevention and Control of Non-Communicable Diseases programme of pulmonary rehabilitation based in the peripheral health centres with support from trained therapists and community volunteers in rural and urban settings.

## Additional material


Online Supplementary Document
