# Supplementary material for: Optimising participation in a pulmonary rehabilitation programme for people living with chronic respiratory diseases in rural India: a feasibility study
Source: J Glob Health. 2025 May 9;15:04143. doi: 10.7189/jogh.15.04143 (PMC12063193; doi:10.7189/jogh.15.04143)
Supplement: Online Supplementary Document [file jogh-15-04143-s001.pdf]

Supplement to: Jebaraj P, Paul B, Isaac R, Reddy SR, Kumar R, Vikas B, Das D, Norrie J, Weller D, Pinnock H; RESPIRE Collaboration. Optimising participation in a pulmonary rehabilitation programme for people living with chronic respiratory diseases in rural India: a feasibility study. J Glob Health. 2025;15:04143.

Table S1: Exercise training

**Exercise Type**	**Modality**	**Average Time (min)**	**Frequency, duration and Intensity of exercise** (Two supervised sessions at the centre, four days of unsupervised sessions)				
			**Week 1**	**Week 2**	**Week 3 & 4**	**Week 5 & 6**	**Week 7 & 8**
**Warm-up**	Global range of Motion exercise	5 – 10	1 set	1 set	1 set	1 set	1 set
**Indoor aerobic Training**	Stepping (1 set of 10 reps)	5 – 15	1 set	2 sets	3 – 4 sets	4 – 5 sets	6 – 8 sets
**Hand exercise**	Squeeze ball (1 set of 20 reps)	5 – 15	1 set	2 sets	3 – 4 sets	4 – 5 sets	6 – 8 sets
**Resistance training for Upper limb**	Free weights for major muscle groups (1 set of 20 reps)	15 – 30	0 kg for 1 set	0.5 kg for 2 sets	1 kg for 2 sets	1 – 1.5 kg for 3 sets	1.5 - 2 kg for 4 sets
**Resistance training for lower limb**	Free weights for major muscle groups (1 set of 20 reps)	15 – 30	0 kg for 1 set	0.5 kg for 2 sets	1 kg for 2 sets	1 – 1.5 kg for 3 sets	1.5 - 2 kg for 4 sets
**Balance training**	Upright positions (1 set of 10 reps)	5 - 15	1 set	2 sets	3 – 4 sets	4 – 5 sets	6 – 8 sets
**Breathing exercise**	Purse lip breathing, Huffing and cuffing technique	5 - 15	1 set	2 sets	3 – 4 sets	4 – 5 sets	6 – 8 sets
**Cool-down**	Stretching	5 - 15	1 set	1 set	1 set	1 set	1 set
**Outdoor Aerobic Training**	Walking (5 days a week)	20 - 30	10 mins of walking	20 mins of walking	80% of the 6-MWT mean walk velocity*		

*Ramanathan RP, Chandrasekaran B. Reference equations for 6-min walk test in healthy Indian subjects (25-80 years). Lung India. 2014;31:35-8.
